# Cytotoxicity, Proapoptotic Activity and Drug-like Potential of Quercetin and Kaempferol in Glioblastoma Cells: Preclinical Insights

**DOI:** 10.3390/ijms251910740

**Published:** 2024-10-05

**Authors:** Magdalena Kusaczuk, Elena Tovar-Ambel, Paola Martín-Cabrera, Mar Lorente, Nélida Salvador-Tormo, Agnieszka Mikłosz, Adrian Chabowski, Guillermo Velasco, Monika Naumowicz

**Affiliations:** 1 Department of Pharmaceutical Biochemistry, Medical University of Bialystok, Mickiewicza 2A, 15-222 Bialystok, Poland; 2 Department of Biochemistry and Molecular Biology, School of Biology, Complutense University and Instituto de Investigación Sanitaria San Carlos IdISSC, 28040 Madrid, Spain; elenatov@ucm.es (E.T.-A.); paolmart@ucm.es (P.M.-C.); mmlorent@pdi.ucm.es (M.L.); nsalvado@ucm.es (N.S.-T.); gvelasco@ucm.es (G.V.); 3 Department of Physiology, Medical University of Bialystok, Mickiewicza 2C, 15-222 Bialystok, Poland; agnieszka.miklosz@umb.edu.pl (A.M.); adrian.chabowski@umb.edu.pl (A.C.); 4 Department of Physical Chemistry, Faculty of Chemistry, University of Bialystok, K. Ciolkowskiego 1K, 15-245 Bialystok, Poland

**Keywords:** quercetin, kaempferol, glioblastoma, apoptosis, cytotoxicity, cell proliferation, drug–membrane interaction

## Abstract

Despite the increasing understanding of the pathogenesis of glioblastoma (GBM), treatment options for this tumor remain limited. Recently, the therapeutic potential of natural compounds has attracted great interest. Thus, dietary flavonoids quercetin (QCT) and kaempferol (KMF) were investigated as potential cytostatic agents in GBM. Moreover, the physicochemical properties of QCT and KMF, determining their bioavailability and therapeutic efficiency, were evaluated. We proved that both polyphenols significantly reduced the viability of GBM cells. We also demonstrated that both QCT and KMF evoked the cytotoxic effect in T98G cells via induction of apoptotic cell death as shown by increased activity of caspase 3/7 and caspase 9 together with an overexpression of the cleaved form of PARP. Apoptosis was additionally accompanied by the activation of stress responses in QCT- and KMF-treated cells. Both polyphenols caused oxidative stress and endoplasmic reticulum (ER) stress, as demonstrated by the increased generation of reactive oxygen species (ROS), deregulated expressions of superoxide dismutases (SOD2 and *Sod1* on protein and transcriptomic levels, respectively), as well as an overexpression of ERO1α, GRP78, p-JNK, and an up-regulation of *Chop*, *Atf4* and *Atf6α* genes. The antitumor effect of QCT and KMF was also confirmed in vivo, showing reduced growth of tumor xenografts in the chick chorioallantoic membrane (CAM) experiment. Moreover, electrophoretic light scattering (ELS) was used to measure the zeta potential of cell membranes upon exposition to QCT and KMF. Additionally, on the basis of existing physicochemical data, the drug-likeness score of QCT and KMF was evaluated. Analyses showed that both compounds accomplish Lipinski’s Rule of 5, and they both fit into the criteria of good central nervous system (CNS) drugs. Altogether, our data support the idea that QCT and KMF might be plausible candidates for evaluation as therapeutic agents in preclinical models of glioblastoma.

## 1. Introduction

Gliomas are the most frequently occurring primary brain tumors of the central nervous system. Out of those, glioblastoma (GBM) is considered the most aggressive and deadliest form of brain cancer. Currently, surgical resection followed by aggressive radio- and chemotherapy is a standard protocol of treatment for GBM patients [1,2]. However, despite the advances in therapeutic strategies, the available treatment options are still ineffective and leave patients with a poor prognosis of survival [2,3]. Thus, new modalities of anticancer drugs are constantly being quested.

Lately, natural therapies have gained much recognition in medicine and pharmacology [4,5,6,7]. Therefore, numerous studies focus on testing the efficacy of natural compounds in an oncological context. To date, it has been demonstrated that phytochemicals with phenolic structures, such as flavonoids, exhibit antiproliferative properties in malignant cells while presenting a good safety profile [4,5,6,7,8,9,10,11]. Among this group of phytochemicals, quercetin (QCT) and kaempferol (KMF) are one of the most widely studied compounds [12,13]. Hence, multiple preclinical studies have shown the cytotoxic effect of both QCT and KMF against, e.g., breast, ovarian, prostate, lung, gastric or hematological cancers [14,15]. However, only a limited amount of research has reported the functioning of these flavonoids in brain malignancies [16,17,18,19,20,21]. Thus, the exact molecular mode of action of QCT and KMF is still insufficiently understood. Michaud-Levesque et al. revealed that QCT significantly decreased the IL-6-mediated STAT3 activation in U87MG and T98G cell lines [22], whereas other reports showed a reduction of X-linked inhibitor of apoptosis protein (XIAP) expression [23], inhibition of the PI3K/AKT pathway [24], or G2-dependent cell cycle arrest [25]. Moreover, Jang et al. demonstrated that an exposition to QCT contributed to the death of the T98G cells via endoplasmic reticulum (ER) stress, Ca^2+^ imbalance, and oxidative stress [26], which is in line with our previous report [16]. Likewise, KMF has been demonstrated to enhance reactive oxygen species (ROS) production, decrease mitochondrial membrane potential, and evoke apoptosis/pyroptosis in GBM cell lines [19,20,27]. Given this, QCT and KMF seem to constitute promising candidates for anti-GBM agents.

Notably, the candidates for potential drugs against GBM must also present good blood-brain barrier (BBB) permeability. Hence, several studies have shown that flavonoids can efficiently penetrate the central nervous system (CNS) [28,29,30,31]. Flavonoids were reported to cross the BBB through carrier-mediated transcellular transport, transcellular diffusion, or paracellular diffusion [32]. Importantly, passive transcellular diffusion is limited only to molecules with sufficient lipophilicity [32]. Therefore, lipophilicity is a first-rate physicochemical parameter describing both pharmacokinetic and pharmacodynamic aspects of drug functioning. The common way of expressing lipophilicity for ionizable compounds is through the logarithm of the n-octanol/water partition coefficient (log*P*). Log*P* values typically fit into a range between −3 (very hydrophilic) and +10 (extremely hydrophobic) [33]. In general, the more lipophilic the compound, the better its ability to pass through the lipidic bilayer of the cell membrane and the higher its intracellular intake [34]. Therefore, the evaluation of liposolubility emerges as a key estimator of drug bioavailability. Another important feature of potential therapeutic agents is their oral bioavailability. Thus, various predictive models which estimate oral bioavailability of drug candidates have been developed. One of the most important and commonly applied rules regarding this issue is Lipinski’s Rule of 5 (Ro5) [34]. The Ro5 assumes that molecules characterized by poor absorption and weak oral permeability have at least two of the following features: a calculated n-octanol/water partition coefficient (*c*log*P*) > 5, a molecular weight (*M*_WT_) > 500, more than 10 acceptor groups (expressed as the sum of O and N atoms), and a number of hydrogen bond donor groups (expressed as the sum of OH and NH groups) exceeding 5 [34].

Interestingly, the surface charge of a cell membrane is also an important biophysical parameter that may reflect the drug–membrane interactions. Considering that the cell-surface charge of the membrane can be affected by drug–membrane binding, this parameter may also serve as a predictor of the membrane-dependent cellular intake of pharmacological agents [35]. The cell-surface charge can be determined by quantification of the cellular electrokinetic potential (zeta potential—*ζ*), which describes the electrical potential of the double layer of the cell-surface. Notably, the molecular composition of the cell membrane may also influence the zeta potential, which makes it an important indicator of membrane-binding mechanisms and cellular uptake in drug delivery [36,37,38,39].

Thus, in this work we investigated the antiproliferative and physicochemical properties of the dietary polyphenols QCT and KMF in human glioma cells. We checked if cellular stress responses might be involved in the process of apoptotic death of GBM cells. Moreover, we used a novel approach based on the measurement of the zeta potential of cell membranes to evaluate the capability of polyphenols to penetrate through the cell membrane. Altogether, our data indicate that these two compounds are good candidates to be investigated as potential therapeutic agents in preclinical models of GBM.

## 2. Results

### 2.1. The Influence of QCT and KMF on Cell Viability

**Figure 1:**
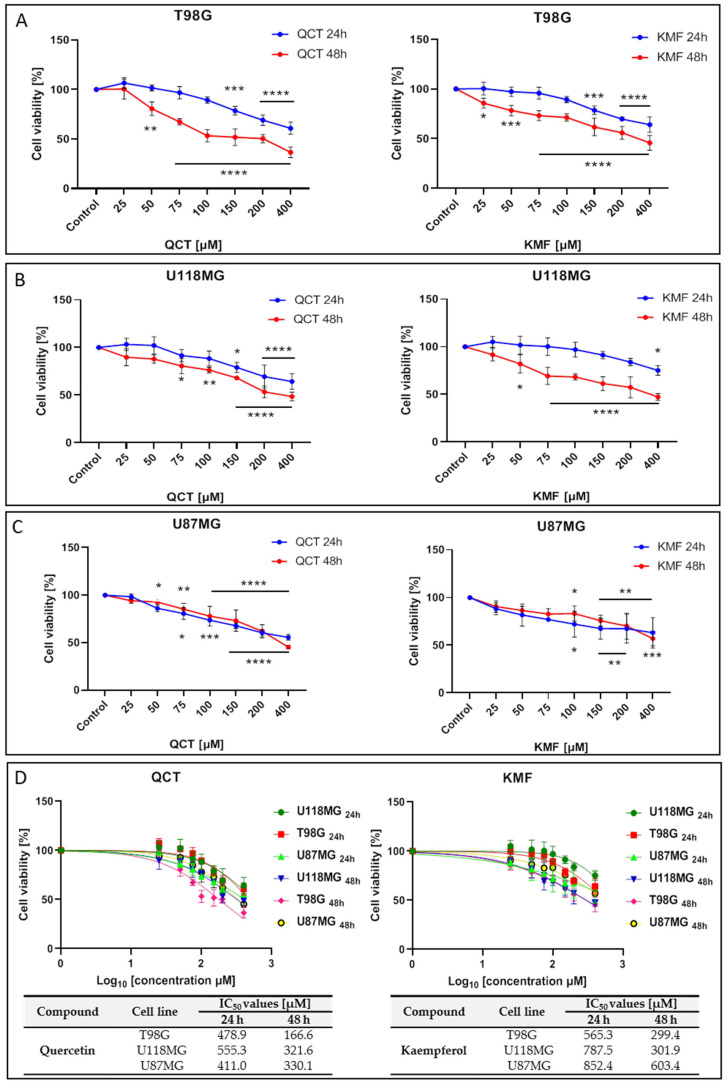
The viability of glioblastoma cells treated with QCT and KMF. Results of the MTT analysis after 24 and 48 h of exposure to QCT and KMF are shown for (**A**) T98G; (**B**) U118MG; (**C**) U87MG cells. (**D**) Cell viability results plotted against logarithmic values of drug concentrations together with a tabulated summary of calculated IC_50_ values are shown for T98G, U118MG, and U87MG cells treated for 24 and 48 h. The results represent means for pooled triplicate values from three independent experiments. Significant alterations are expressed relative to controls and marked with asterisks. Statistical significance was * *p* ≤ 0.05; ** *p* ≤ 0.005; *** *p* ≤ 0.0005; **** *p* ≤ 0.0001.

To analyze the effects of QCT and KMF on the viability of human glioma cell lines T98G, U118MG and U87MG, the MTT assay was performed. In this respect, a concentration range of 25–400 μM was used for both polyphenols and cells were treated for 24 h and 48 h. Treatment of all tested cell lines with both QCT and KMF resulted in dose- and time-dependent decreases in cell survival (Figure 1). Both polyphenols diminished the viability of T98G cells to a similar extent, reaching up to 60% of unviable cells after 48 h of incubation (Figure 1A). Likewise, in U118MG cells, QCT and KMF evoked antiproliferative effects with an overall 60% drop of viable cells in the highest tested dosage of flavonoids after 48 h of treatment (Figure 1B). Analogical effects were observed in the case of U87MG cells; however, there was a less pronounced decrease in viability (approx. 50% for QCT and 40% for KMF) (Figure 1C). For all tested cell lines, more pronounced cytotoxicity of QCT and KMF was observed after 48 h of incubation in comparison to 24 h of treatment (Figure 1A–C). The IC_50_ values for each compound were calculated using GraphPad Prism 8.0.1 software (Figure 1D). Based on the MTT results and the IC_50_ values, the T98G cells were selected for further examinations, and concentrations of 100 and 200 μM were chosen to perform a detailed analysis of the molecular mode of action of QCT and KMF.

### 2.2. The Effect of QCT and KMF on Zeta Potential of Cell Membranes

**Figure 2:**
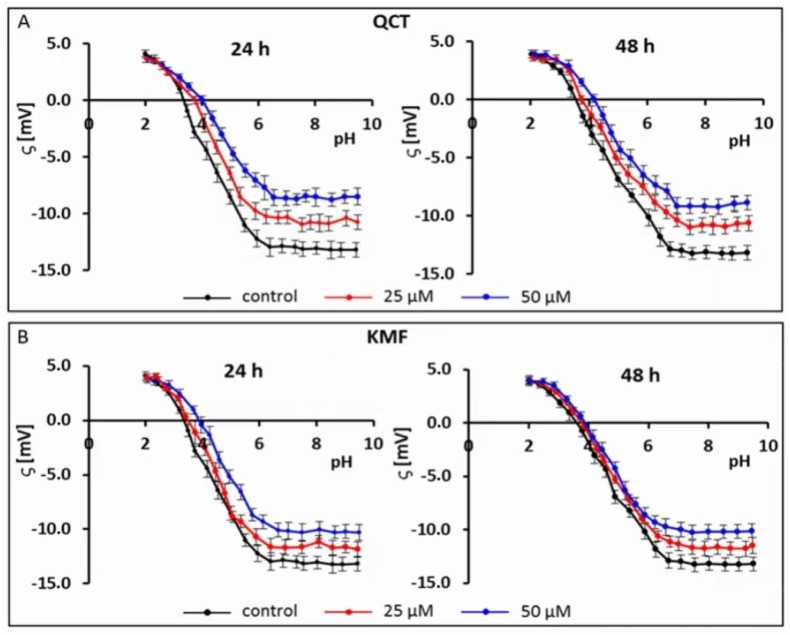
Typical pH-dependence of the zeta potential of human glioblastoma T98G cells treated with (**A**) QCT; (**B**) KMF for 24 and 48 h.

Recently, electrophoretic light scattering (ELS) has occurred as a promising approach for recording the zeta potential of animal cells. Given this, the ELS technique has already been applied in our previous works to study the cell-surface charge of GBM cells treated with phenolic compounds [40,41]. Based on the results of the MTT assay and the calculated IC_50_ values, the sub-lethal concentrations of 25 and 50 μM of QCT and KMF were selected for this experiment to avoid apoptosis-related changes in the membrane of T98G cells. Hence, to check if polyphenols interact with/adsorb on the cell membrane, the microelectrophoretic mobility measurements were undertaken for cells treated with 25 and 50 μM concentrations of QCT and KMF (Figure 2; Table S1).

The pH-dependent changes in the zeta potential of T98G cells treated with both QCT or KMF are described by similarly shaped curves (Figure 2A,B). With a decrease in pH, the values of the positive zeta potential of membranes increased, but only to a certain extent. Conversely, along with an increase in pH values, the negative zeta potential increased up to a plateau. Moreover, it was found that in low pH values, the presence of flavonoids did not affect the *ζ* values. However, with the increasing pH values, a substantial increase (i.e., less negative) in the zeta potential of the above-mentioned cell membranes upon QCT and KMF treatment was observed in comparison to the values achieved for untreated T98G cell membranes (Figure 2A,B; Table S1). The data presenting the values of the zeta potential for selected pH (pH~2 and pH~9) together with the isoelectric point values for cell membranes of tested cells are placed in Table S1 (Supplementary Material). The isoelectric point of GBM cell membranes treated with QCT or KMF shifted to higher pH values compared to the untreated cells (Table S1). This effect could be due to a higher accumulation of basic groups in the membrane of QCT-and KMF-treated T98G cells. These results may indicate that in lower pH of the environment the QCT and KMF can pass through the cell membrane.

With the aim of performing a more in-depth evaluation of the drug-likeness of QCT and KMF as CNS-active agents, we subsequently contrasted our experimental results with the already existing data on the key physicochemical parameters of these flavonoids. Although the presented values might differ slightly depending on the calculation method, the results available across the literature fit into the range of the theoretical criteria for CNS-drugs. As such, a compilation of the QCT and KMF physicochemical constants in comparison to the theoretical values of physicochemical characteristics of CNS-drugs was listed in Table 1.

### 2.3. The Effect of QCT and KMF on Cell Proliferation and Apoptosis

**Figure 3:**
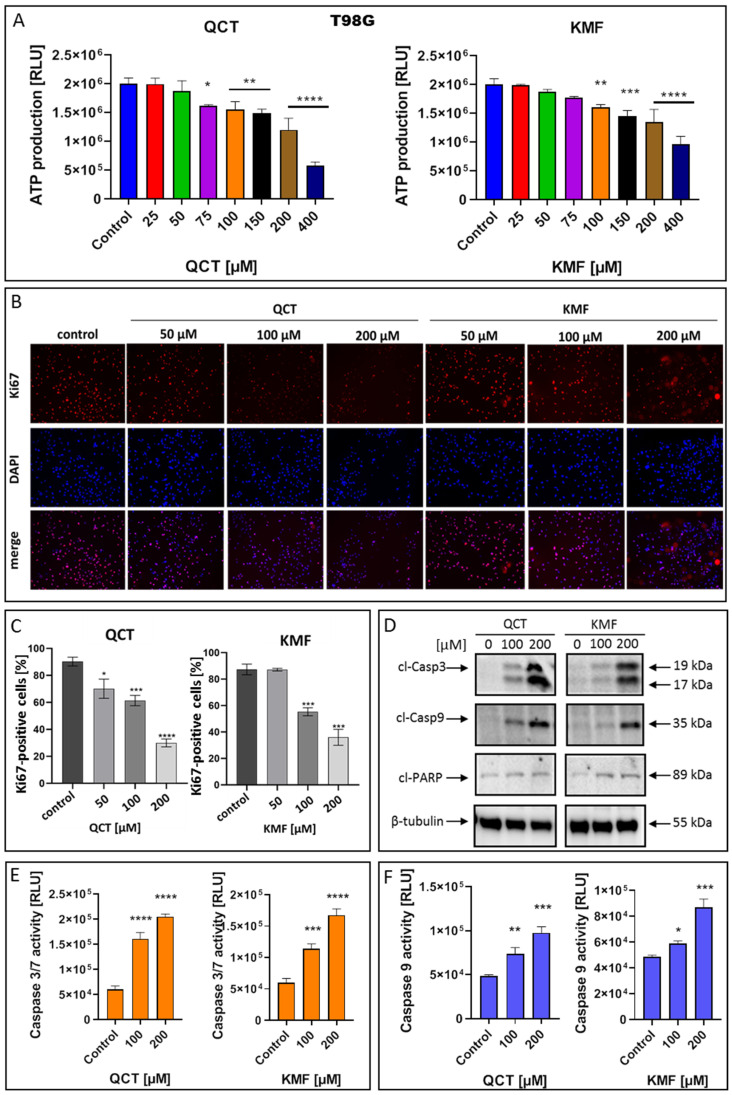
The effect of QCT and KMF on proliferation and apoptosis of GBM cells. (**A**) The ATP production in T98G cells upon treatment with QCT and KMF for 48 h; the immunofluorescence microscopy of Ki67-immunostained T98G cells. The Ki67-staining and the DNA counterstain with DAPI was performed for cells cultured for 48 h. (**B**) Representative images visualized under ZAISS-Axioplan 2 fluorescence microscope are shown (magnification ×20). (**C**) Bar graph illustrating the number of Ki67-immunolabeled T98G cells. (**D**) Representative Western blot images showing expressions of apoptosis-related proteins in T98G cells treated with QCT and KMF for 48 h; (**E**) Caspase 3/7 activity in T98G cells exposed to QCT and KMF for 48 h. (**F**) Caspase 9 activity in T98G cells exposed to QCT and KMF for 48 h. Significant alterations are expressed relative to controls and marked with asterisks. Statistical significance was * *p* ≤ 0.05; ** *p* ≤ 0.005; *** *p* ≤ 0.0005; **** *p* ≤ 0.0001.

To confirm that QCT and KMF can enter the cells and cause depletion in ATP production, the CellTiter Glo assay was performed in T98G cells treated with these polyphenols for 48 h (Figure 3A). Indeed, the content of ATP in QCT- and KMF-treated cells was reduced. Significant reductions in ATP levels were detected in the concentrations of polyphenols starting from 75 μM and 100 μM for QCT and KMF, respectively (Figure 3A). To confirm the influence of QCT and KMF on the proliferation of glioblastoma cells, Ki67/DAPI staining was performed (Figure 3B,C). The Ki67 is expressed in the nucleus during the whole cell cycle except for the G0 phase. Thus, Ki67-positive cells are indicative of active proliferation. Figure 3B shows the representative images of Ki67-immonostained cells, DAPI-stained nuclei and merged images. The T98G cells showed reduced proliferation along with increasing concentrations of QCT and KMF (Figure 3C). These results indicate that both polyphenols caused cytotoxic and cytostatic effects in T98G cells. To further evaluate whether the cytotoxicity of QCT and KMF resulted from the apoptotic cell death, the markers of this process were evaluated. Thus, T98G cells were assayed for evidence of caspase-dependent apoptosis. In this respect, the enzymatic activity of caspases 3/7 and 9, together with the expression levels of the cleaved forms of caspase 3 (cl-Casp3), caspase 9 (cl-Casp9), and PARP (cl-PARP) were evaluated (Figure 3D–F). The Western blot analysis showed a significant overexpression of cl-Casp3 and cl-Casp9 followed by PARP cleavage in T98G cells upon treatment with both polyphenols (Figure 3D). The more pronounced effect was observed in the case of higher tested concentrations of QCT and KMF (200 μM) suggesting a dose-dependent mode of action. These results were confirmed by the luminescent assay. Indeed, stimulation with both QCT and KMF markedly elevated the activity of caspase 3/7 (Figure 3E) as well as caspase 9 (Figure 3F) in T98G cells. Altogether, these results suggest that apoptotic death might be responsible for the elimination of GBM cells upon treatment with QCT and KMF.

### 2.4. The Effect of QCT and KMF on Stress Responses

**Figure 4:**
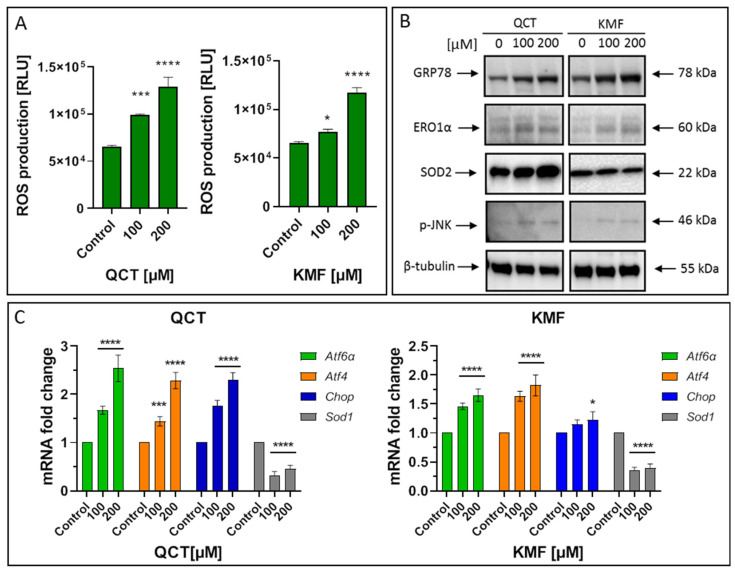
(**A**) The effect of QCT and KMF on stress responses in glioblastoma T98G cells. ROS levels in cells treated with QCT and KMF for 48 h. (**B**) Representative Western blot images showing expression levels of proteins connected with ER stress and oxidative stress in cells treated with QCT and KMF for 48 h. (**C**) Real-time qPCR analysis of *Atf6α*, *Atf4*, *Chop*, and *Sod1* gene expression in cells treated with QCT and KMF for 48 h. Results are shown as relative fold change in mRNA expression in comparison to control, where the expression level was set as 1. Significant alterations are expressed relative to controls and marked with asterisks. Statistical significance was * *p* ≤ 0.05; *** *p* ≤ 0.0005; **** *p* ≤ 0.0001.

To identify the possible complementary mechanisms supporting the proapoptotic effect of QCT and KMF in GBM cells, we decided to quest for the initiation of stress-dependent pathways (Figure 4). Since the pro-oxidant activity of polyphenols has already been well documented [16,52], we sought to determine if oxidative stress might be initiated upon exposure to QCT and KMF. Thus, ROS production was evaluated by the luminescent assay (Figure 4A). We established that H_2_O_2_ levels significantly increased in T98G cells treated with both tested concentrations of polyphenols (Figure 4A). Accordingly, the expression of the antioxidant enzymes was significantly deregulated (Figure 4B,C). As such, QCT caused an increase in SOD2 expression and down-regulated *Sod1* (Figure 4B,C). Interestingly, ROS production in QCT-treated cells was elevated despite increased levels of SOD2, which might indicate that the antioxidant systems may be insufficient to compensate for the overall ROS generation and prevent the redox imbalance. In contrast, in the case of KMF, no visible increase in SOD2 expression, together with down-regulated levels of *Sod1*, was observed (Figure 4B,C). These results might suggest that oxidative stress may be activated upon exposure of T98G cells to QCT and KMF. Furthermore, since the connection between oxidative stress and ER stress is already well-established [2], we investigated whether ER stress was also activated in GBM cells upon treatment with QCT and KMF. One of the key proteins linking oxidative stress and ER stress is the endoplasmic reticulum oxidase 1α (ERO1α), a key player in redox protein folding in the ER [2]. Indeed, both QCT and KMF treatment resulted in an overexpression of ERO1α in T98G cells, suggesting that oxidative stress might be accompanied by the ER stress (Figure 4B). In this respect, key markers of the ER stress have been evaluated (Figure 4B,C). The exposition of T98G cells to QCT and KMF resulted in an overexpression of GRP78—a molecular chaperone responsible for guarding the ER homeostasis and initiating the unfolded protein response (UPR) (Figure 4B) [2]. Accordingly, other mediators of the UPR cascade downstream of the GRP78 signaling, such as *Atf4*, *Atf6α* and *Chop*, were also significantly up-regulated (Figure 4C). Moreover, the expression of the phosphorylated form of stress-induced kinases (p-JNK), known to activate multiple signaling routes, including the proapoptotic pathway, was also increased in the case of both QCT and KMF (Figure 4B). Interestingly, stimulation with KMF resulted in marked up-regulation of proapoptotic *Chop* only in the case of higher 200 μM concentration of this polyphenol (Figure 4C). This might indicate that although both polyphenols may act as proapoptotic agents causing cellular stresses in GBM cells, QCT may present higher anti-GBM potential than KMF. Nevertheless, further analyses are necessary to elucidate the full molecular profile of QCT- and KMF-dependent pathways in GBM cells.

### 2.5. The Effect QCT and KMF on Inhibition of Tumor Growth In Vivo

**Figure 5:**
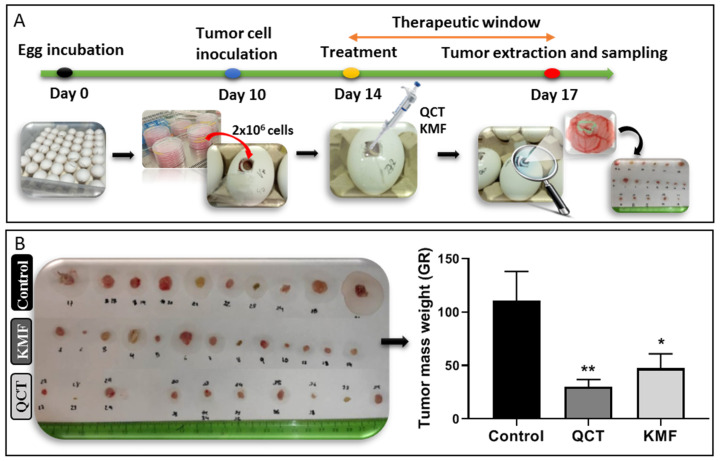
(**A**) Chicken embryo in vivo assay. Time schedule of the chicken embryo in vivo experiment. (**B**) Weight of control tumors vs. tumors treated with 200 μM QCT and 200 μM KMF. Significant alterations are expressed relative to controls and marked with asterisks. Statistical significance was * *p* ≤ 0.05; ** *p* ≤ 0.005.

Taking into consideration the promising results of the in vitro analyses, the antiproliferative potential of QCT and KMF was evaluated in a more complex environment. Thus, a chicken embryo model-based in vivo assay was performed (Figure 5). The time course of the CAM experimental procedure is shown in Figure 5A. Results of the analysis showed that both QCT and KMF decreased the size of tumors (Figure 5B). However, tumors treated with QCT tend to show higher inhibition of tumor mass growth than those treated with KMF (Figure 5B), which is consistent with the rest of the in vitro results. Altogether, the weight of tumors treated with QCT and KMF was significantly reduced in comparison to controls, confirming the antiproliferative capacity of both polyphenols in vivo.

## 3. Discussion

Glioblastoma (GBM) is considered one of the most abundant types of glial tumors with a poor prognosis of survival. Despite the extensive efforts to treat this malignancy, currently available therapeutic options are ineffective in curing GBM. Thus, new modalities of anticancer drugs are required to advance the treatment of brain tumors [2]. Lately, natural substances have been widely recognized as promising anticancer agents [16,25,26,39,41,53,54,55,56,57]. One such class of compounds is plant polyphenols. Out of this group, quercetin (QCT) and kaempferol (KMF) are already well-established to possess anticancer activity [55,57]. Given this, QCT and KMF have been demonstrated to induce autophagy, cause cell cycle arrest, decrease mitochondrial membrane potential, reduce cell migration and angiogenesis, and evoke apoptosis in various cellular models of cancer, e.g., cholangiocarcinoma, leukemia, lung, prostate, gastric, breast or colon cancer [15,55,56]. However, although a substantial number of studies exploring the influence of QCT and KMF on a variety of cancer cell lines exist, the molecular mechanisms initiated upon exposure to both these compounds in GBM are still insufficiently understood.

As such, we investigated the cytotoxic effect of QCT and KMF on T98G, U118MG and U87MG glioblastoma cell lines. We demonstrated that both polyphenols significantly reduced the viability of tested cells, which is consistent with several previous reports showing the cytotoxic effect of QCT and KMF in GBM in vitro [17,19,57]. Moreover, preliminary reports suggest that QCT and KMF can cross the BBB, emerging as potentially attractive agents for GBM treatment [30,58]. On these premises, we decided to evaluate whether QCT and KMF present good membrane permeability and high CNS drug-like potential using both experimental and theoretical approaches. We demonstrated that the zeta potential of cells treated with both polyphenols was almost intact in low pH, indicating that neither QCT nor KMF adsorbed on the cell-surface. The influence of the pH on drug intake may be specifically important in terms of cancer cells, as transformed cells show more acidic extracellular pH in comparison to their normal counterparts [59,60,61,62]. Furthermore, based on the analysis of experimental data, the guidelines for the physical properties of compounds have been developed and used to derive computational algorithms to predict CNS efficacy. Such analyses are often conjunct with the Ro5 and serve as predictors of the pharmacological utility of potential drugs. In our work, we listed and analyzed the key physicochemical parameters of QCT and KMF, demonstrating that both polyphenols fulfill the criteria of the Ro5, having molecular weight ≤ 500, clogP ≤ 5, number of hydrogen bond acceptors (O) ≤ 10 and number of hydrogen bond donors (OH, NH) ≤ 5. Meeting the Ro5 criteria indicates that both polyphenols may be an orally bioavailable drugs, which is particularly important for substances for which dietary consumption is the most natural way of intake. Notably, QCT and KMF fulfill most of the restricted physicochemical requirements for the potential CNS drugs, such as molecular weight, calculated n-octanol/water partition coefficient, distribution coefficient, acid dissociation constant, number of rotatable bonds, and number of H-bond acceptors [42,48]. Moreover, computational analyses showed that both QCT and KMF presented beneficial Drug-Likeness Model Score, reaching 1.64 and 0.91 respectively [43]. As such, both phytochemicals show good membrane permeability, oral bioavailability, and display desirable CNS drug-likeness properties, which certainly encourages their further examination as anti-GBM agents.

**Figure 6:**
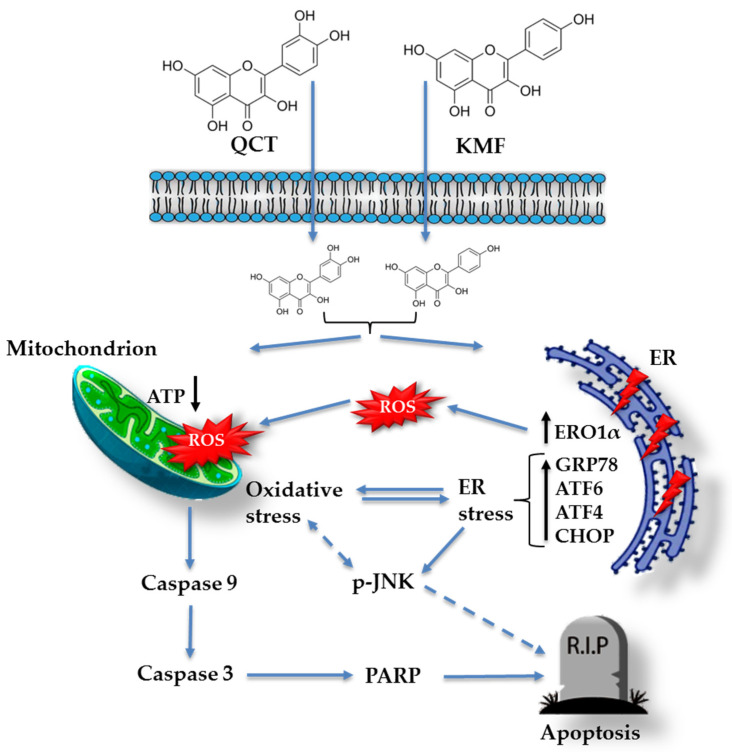
Tentative model of QCT and KMF mode of action in glioblastoma cells. Continuous arrows symbolize well-established molecular pathways. Dashed arrows show possible indirect interactions.

Based on these data, we subsequently investigated the possible mechanism underlying the cytotoxic effect of QCT and KMF. We demonstrated that treatment with both polyphenols resulted in a significant reduction of ATP production, decreased the proliferation rate, and finally, caused apoptotic death of T98G cells. Apoptosis was accompanied by an elevated activity of caspase 3/7 and caspase 9 as well as an overexpression of cl-Casp3, cl-Casp9 and cl-PARP, which is consistent with previous reports [19,25,27,63,64,65,66]. Taking into consideration that polyphenols might act as antioxidants as well as pro-oxidants depending on the time of exposure and the dosage [52,67], the engagement of stress responses activated upon stimulation with QCT and KMF has been evaluated. Indeed, both QCT and KMF elevated the levels of ROS production in T98G cells. The overproduction of ROS may disrupt the balance between oxidative and antioxidant systems, resulting in reduced antioxidant capacity. Indeed, excessive ROS generation was accompanied by deregulated expression of key antioxidant enzymes SOD2 and *Sod1*. However, changes in the expression of antioxidant enzymes were dependent on the polyphenol used. Although both QCT and KMF down-regulated the expression of *Sod1*, the SOD2 was increased upon treatment with QCT but not in cells stimulated with KMF. These findings are consistent with the results of our previous studies, where we showed increased expression of SOD2 and unchanged/decreased expression of SOD1 upon stimulation with QCT [16]. On the other hand, the results for KMF are in partial agreement with those reported by Sharma et al., who demonstrated decreased expression of SOD1 in KMF-treated U87MG and T98G cells [27]. Moreover, since stress responses intercross and merge with each other, we decided to check if oxidative stress might be accompanied by the ER stress. Thus, ERO1α, a key enzyme in redox protein folding in the ER, was assessed [68]. The expression of ERO1α was augmented upon stimulation with QCT and KMF. Moreover, both polyphenols promoted an overexpression of the hallmark molecules of the ER stress, such as GRP78, p-JNK, *Atf4*, *Atf6α* and *Chop*. A tentative model of QCT- and KMF-dependent apoptosis in GBM cells is depicted in Figure 6. To finally confirm the promising results of the in vitro analyses, QCT and KMF were tested in the in vivo setting. The CAM experiment showed that both polyphenols inhibited tumor growth in vivo. Similar results were reported by Chen et al. who demonstrated a reduced mass of xenografted U87MG tumors implanted subcutaneously in nude mice after treatment with QCT [17]. Likewise, KMF was demonstrated to suppress GBM xenograft growth in BALB/c nude mice [19].

According to our knowledge this is one of the few reports showing not only the engagement of the oxidative stress but also the ER stress in the apoptotic death of glioblastoma cells. Moreover, a novel approach based on the measurement of the zeta potential of cell membrane has shown that acidic extracellular milieu facilitates transfer of polyphenols through the cell membrane. These findings warrant further investigations concerning selection of the appropriate co-therapeutic strategies targeted at modulation of cellular stress responses and facilitating cellular up-take of polyphenols. Altogether, although our data indicate that QCT and KMF may be potentially good candidates for anti-GBM drugs, numerous studies should be undertaken to confirm their therapeutic potential in patients.

## 4. Materials and Methods

### 4.1. Reagents

DMEM containing glucose at 4.5 mg/cm^3^ with GlutaMax (31966021), penicillin 10,000 U/mL-streptomycin 10,000 µg/mL (15140-122), PBS (14190-144), and Fetal Bovine Serum (FBS) Gold (A5256701) were provided by Thermo Fisher Scientific (Waltham, MA, USA). The 0.05% trypsin 0.02%–EDTA (P10-027100) was provided by PAN Biotech (Aidenbach, Germany). The dimethyl sulfoxide (DMSO; 25-950-CQC) was from Corning (Manassas, VA, USA). The methylthiazolyldiphenyl-tetrazolium bromide (MTT, M2128), quercetin (≥95.0% (HPLC); Q4951), kaempferol (≥97.0% (HPLC); 60010), Mowiol^®^ 4-88 (81381), and the TRIzol Reagent were provided by Sigma-Aldrich (St Louis, MO, USA). The CellTiter-Glo luminescent cell-viability assay (G7570), the Caspase-Glo 3/7 assay (G8981), the Caspase-Glo 9 assay, and the ROS-Glo H_2_O_2_ system (G8820) were purchased from Promega (Fitchburg, WI, USA). The Protease/Phosphatase Inhibitor Cocktail (#5872S), the monoclonal rabbit anti-GRP78 (#3183), anti-ERO1α (#3264), anti-Caspase 9 (cleaved; #20750), and the polyclonal anti-β-tubulin (#2146) antibodies were provided by the Cell Signaling Technology (Boston, MA, USA). The monoclonal rabbit anti-PARP (cleaved; A22535), anti-Casp 3 (cleaved; A11021), anti-p-JNK (phosphorylated, AP0631) and anti-SOD2 (A19576) antibodies were ordered from ABClonal (Woburn, MA USA). The radioimmunoprecipitation assay (RIPA; 89900) lysis buffer, the HRP-conjugated anti-rabbit IgG (#7074), the BCA protein-assay kit (23225), and the rabbit monoclonal anti-Ki67 antibody (RM-9106) were purchased from Thermo Fisher Scientific. The Clarity Western ECL Substrate (#1705061), the Laemmli buffer (#1610737), and the Criterion TGX Stain-Free Precast Gels (#5678035) were supplied by Bio-Rad (Hercules, CA, USA). The EvoScript Universal cDNA Master kit and the FastStart Essential DNA Green Master were provided by Roche Molecular Systems (Pleasanton, CA, USA).

### 4.2. Cell Culture and Exposure to Flavonoids

The T98G [CRL-1690], U118MG [HTB-15] and U87MG [HTB-14] cell lines were purchased from American Type Culture Collection (ATCC, Manassas, VA, USA) and cultured as described in our previous works [16,40]. A non-tumorigenic (T98G) and tumor-forming cells (U118MG and U87MG) were selected for the study. Briefly, cells were grown in DMEM containing 10% FBS, 4.5 mg/mL glucose, 100 U/mL penicillin, 100 μg/mL streptomycin, and 2 mM L-glutamine. Cells were cultured in Falcon flasks (BD Biosciences) in a 5% CO_2_ incubator (Galaxy S+; Eppendorf, Hamburg, Germany) at 37 °C. Cells reaching confluence were seeded into 96-well plates (Nunclon, Thermo Fisher Scientific), 6-well plates (Thermo Fisher Scientific) or 100 mm dishes (Thermo Fisher Scientific) dependent on the experiment, and growth medium was substituted with DMEM containing various concentrations of QCT or KMF. Cells were further incubated for 24 or 48 h. QCT and KMF were dissolved in dimethyl sulfoxide (DMSO) as 20 mM stock solutions and subsequently diluted into micromolar concentrations with growth media, keeping the final concentration of DMSO < 1% in culture. For the cell-based experiments control cells were supplemented with the vehicle (DMSO) in the concentration of 0.5% in a culture medium. After incubation, cells were subjected to further analyses.

### 4.3. MTT Analysis

The MTT test was performed according to the protocol described in detail in our previous works [16,40]. Briefly, T98G, U118MG and U87MG cells were seeded in 96-well plates at a density of 3 × 10^3^ cells/well and then cultured with QCT and KMF at concentrations of 25–400 μM for 24 and 48 h. Next, cells were washed twice with PBS and incubated with 200 μL MTT solution (0.25 mg/mL in PBS) at 37 °C in a humidified 5% CO_2_ atmosphere for 3 h. The medium was removed, and formazan products were solubilized in 200 μL of 0.1 mM HCl in absolute isopropanol. The absorbance of a converted dye in living cells was read on a microplate reader (Rayto, Rayto Life and Analytical Sciences Co., Ltd., Shenzhen, China) at a wavelength of 570 nm. The viability of polyphenols-treated cells was calculated as a percentage of control untreated cells.

### 4.4. Electrophoretic Light Scattering Measurements

The electrophoretic mobility of GBM cells was measured with a zeta potential analyzer (Zetasizer Nano ZS; Malvern Instruments Ltd., Malvern, UK) using the electrophoretic light scattering (ELS) technique. Disposable folded capillary cells (Malvern DTS 1070) were used to perform the experiment. All measurements were carried out as a function of pH using a WTW InoLab pH 720 laboratory meter (WTW, Weinheim, Germany).

The samples suspended in 0.155 M NaCl solution were titrated to the desired pH (ranging from 2 to 9.5) with sodium hydroxide or hydrochloric acid, and measurements of *ζ* were done every ± 0.3 pH units. Six electrophoretic mobility measurements (each covering 100–200 series with duration of 2 s), for every sample, at a given pH value were performed.

The zeta potential of the cells was calculated according to the approach described in our previous papers [39,40] from the electrophoretic mobility using Henry’s equation:

$$\zeta =\frac{3\cdot \eta \cdot \mu }{2\cdot \varepsilon \cdot \varepsilon_{0}\cdot f\left(\kappa a\right)}$$

where: *µ* is the electrophoretic mobility, *η* is the viscosity of the aqueous solution, *ε* is the relative permittivity of the medium, $\varepsilon_{0}$ the permittivity of free space, and ƒ(κa) is Henry’s function.

### 4.5. CellTiter-Glo Assay

A measurement of the cellular ATP levels in the control and QCT/KMF-treated T98G cells was performed using the CellTiter-Glo assay following the supplier’s specifications. Briefly, T98G cells were seeded in white-walled 96-well culture plates (Nunclon) at a density of 1 × 10^3^ cells per well. Cells were allowed to attach and then incubated with a medium containing QCT or KMF at concentrations of 25–400 μM for 48 h. After incubation, 100 μL of staining solution (CellTiter-Glo reagent) was added to each well and mixed at 300 rpm on an orbital plate shaker for 2 min to induce cell lysis. Cells were incubated at room temperature for 10 min to stabilize the luminescence signal. The signal was then recorded using the microplate reader (FLUOstar Omega, BMG LABTECH, Ortenberg, Germany).

### 4.6. Ki67/DAPI Staining

The Ki67 staining is frequently used in oncology to estimate a tumor proliferation index. In order to perform this staining, T98G cells were seeded at a density of 3 × 10^4^ cells per well in 24-well plates (Thermo Scientific) with glass slides covers of 10 mm diameter on the bottom of each well. Next, control cells and cells treated with QCT and KMF in the concentrations of 50 μM, 100 μM and 200 μM were cultured for 48 h. After incubation, cells were fixed with 4% *p*-formaldehyde for 15 min at room temperature and washed tree times with PBS. Subsequently, cells were blocked and permeated with 10% goat serum and 0.25% Triton X-100 in PBS (PBS-T) for 1 h at room temperature. Afterwards, cells were incubated with the primary antibody Ki67 (1:300) at 4 °C overnight. On the next day, the cells were washed with PBS-T and incubated with the corresponding secondary antibody conjugated to Alexa-594 dye (Invitrogen, Waltham, MA, USA) at a dilution of 1:500 for 1 h at room temperature in the dark. The nuclei were stained with DAPI (1:5000), which was added simultaneously with the secondary antibody. After the washing of the secondary antibody, the preparations were assembled with Mowiol 4–88 (a water-soluble hydrocolloid mucoadhesive based on poly (vinyl alcohol)) and visualized under the ZAISS-Axioplan 2 fluorescence microscope (Zaiss, Göttingen, Germany) using the Metamorph-Offline 6.2 software (Molecular Devices LLC, San Jose, CA, USA). Cells from five random fields were examined at ×20 magnification, and the percentage of fluorescent-positive cells/DAPI-positive cells in each field was measured. A quantitative analysis of Ki67(+)cells/DAPI(+) cells ratio was performed, and the results were expressed as a number of Ki67-positive cells compared to the control.

### 4.7. Caspase 3/7 and Caspase 9 Activity

Measurement of caspase 3/7 and caspase 9 activities after exposure to QCT and KMF was performed using the luminescent Caspase-Glo 3/7 and Caspase-Glo 9 assays (Promega), respectively. The methodology was executed following the manufacturer’s instructions. Briefly, T98G cells were seeded in white-walled 96-well culture plates (Nunclon) at a density of 1 × 10^4^ cells/well. Subsequently, the cells were incubated with a medium containing QCT and KMF at concentrations of 100 and 200 μM for 48 h. After incubation, 100 μL of Caspase-Glo 3/7 or Caspase-Glo 9 reagent was added to each sample. The cells were mixed using a plate shaker at 300 rpm for 45 s and left in the dark at room temperature for 40 min. The incubation was followed by measurement of the luminescence on a microplate reader (FLUOstar Omega, BMG LABTECH, Ortenberg, Germany).

### 4.8. Reactive Oxygen Species Generation

Generation of ROS was detected using the luminescent ROS-Glo H_2_O_2_ assay according to the supplier’s specifications. A detailed procedure is described in our previous work [16]. Briefly, T98G cells were plated at a density of 1 × 10^4^ per well in 80 μL of DMEM in 96-well white-walled plates (Nunclon). Then, the DMEM was replaced with a medium containing 100 and 200 μM QCT and KMF for 48 h. The substrate solution was added to the cells, which were then cultured for an additional 6 h. Subsequently, 100 μL of ROS-Glo detection solution was added to each well, and the relative luminescence units (RLU) were recorded using a microplate reader (FLUOstar Omega, BMG LABTECH, Ortenberg, Germany).

### 4.9. RNA Isolation and Gene Expression Analysis

The procedure of the RNA purification and further RT q-PCR analysis was described in our previous works [16,69]. Briefly, total RNA was isolated using the TRIzol Reagent (Sigma-Aldrich) with the DNase I treatment according to the manufacturer’s instructions. Spectrophotometric measurements (A260/A280) were carried out to evaluate the quantity and quality of the extracted RNA (NanoDrop 2000, Thermo Scientific). Afterwards, the RNA was reverse-transcribed into cDNA using the EvoScript Universal cDNA Master kit (Roche Molecular Systems) using 0.5 μg of purified total RNA in 20 μL of the reaction mixture. Amplification of the product was performed using the FastStart Essential DNA Green Master (Roche Molecular Systems), and the fluorescent signal was detected on the LightCycler 96 System Real-Time thermal cycler (Roche, Mannheim, Germany). The following reaction parameters were applied: initial denaturation at 94 °C for 10 min, followed by 45 cycles of denaturation at 94 °C for 15 s, annealing at 57 °C–61 °C for 15 s, and extension at 72 °C for 15 s. Primer sequences for *Atf4*, *Atf6α*, *Sod1*, and *Chop* and the housekeeping *Rpl13a* have been described in previous works [16,70,71]. Gene expression was analyzed using the relative quantification method [16].

### 4.10. Protein Assay and Immunoblotting

A standard Western blotting procedure was used to detect protein content in total lysate [69]. In brief, lysates of T98G cells were prepared using ice-cold RIPA buffer containing a mix of protease and phosphatase inhibitors. The total protein concentration was assayed using the BCA method with bovine serum albumin (BSA) as a standard. Next, lysates were reconstituted in Laemmli buffer (Bio-Rad), and equal amounts of the proteins (20 µg per sample) were loaded on the Criterion TGX Stain-Free Precast Gels (Bio-Rad) for sodium dodecyl-sulfate polyacrylamide gel electrophoresis (SDS-PAGE). Size-separated proteins were transferred onto polyvinylidene difluoride (PVDF) membranes. After blocking in 5% non-fat dry milk for 1 h, membranes were incubated overnight at 4 °C with the corresponding primary antibodies, i.e., anti-cl-Casp3 (1:1000), anti-cl-Casp9 (1:1000), anti-cl-PARP (1:1000), anti-GRP78 (1:1000), anti-ERO1α (1:1000), anti-p-JNK (1:1000), anti-SOD2 (1:1000). The β–tubulin was used as a loading control. Thereafter, bound antibodies were detected with suitable anti-rabbit IgG horseradish peroxidase-conjugate secondary antibodies (1:3000). The protein bands were imaged by chemiluminescence using the Clarity Western ECL Substrate (Bio-Rad). The Western blot assay was performed on samples from two independent experiments.

### 4.11. Chick Chorioallantoic Membrane (CAM) Model

To evaluate tumor growth in vivo the CAM experiment was performed. For the CAM xenografts, premium, specific pathogen-free, fertilized chicken eggs were used. To form xenografts, the U87MG cells were applied. In this respect, fertilized chicken eggs were incubated for 10 days at 37 °C and 55% humidity, rotating every 2 h. On day 10 of the development, a small window was made in an eggshell over the air sack in a laminar hood cabinet. Next, small silicone rings were placed on the chorioallantoic membrane, in which 50 µL of cell suspension was inoculated over a scratched vein of the chicken embryo. The cell suspension contained 2 × 10^6^ of U87MG cells mixed in a 1:1 ratio with matrigel. Treatment was applied by putting 50 µL of culture medium containing 200 µM QCT or KMF three days before the opening. Control eggs were injected with the vehicle. Eggs were then incubated with treatment for 72 h. Finally, after incubation (day 17 of the experiment), tumors were resected from the eggs, weighted, and measured. For each experimental condition 20 eggs were used. The scheme of the course of the experiment is presented in Figure 5.

### 4.12. Statistical Analysis

Experiments were replicated in triplicate, and data were expressed as means ± standard deviation (SD). GraphPad Prism 8.0.1 software (GraphPad Software, Inc., Boston, MA, USA) was applied for statistical analysis. A one-way analysis of variance (ANOVA) was carried out for comparisons between control and treated groups. The half maximal inhibitory concentration (IC_50_) values were calculated through a non-linear regression using the GraphPad Prism 8.0.1 software. The ELS results were reported as means ± SD from three independent measurements and analyzed using one-way ANOVA with Scheffe’s F test. A * *p* ≤ 0.05; ** *p* ≤ 0.005; *** *p* ≤ 0.0005; **** *p* ≤ 0.0001 was considered statistically significant.

## 5. Conclusions

Although our findings suggest that QCT and KMF may be potentially good candidates for anti-GBM drugs, there are several limitations that need to be addressed. One of the principal issues encountered in the in vitro studies is the dosage of the applied treatments. Very often, and also in the case of our study, these doses highly exceed concentrations achievable in vivo and cannot directly reflect the expected effect in living organisms. Thus, there is still a need to perform animal-based studies to get more comprehensive data on the metabolism, pharmacokinetics, and possible adverse effects of QCT and KMF. Moreover, despite the optimistic results of the preclinical studies, natural phenolic compounds per se present a lot of limitations to be successfully translated into clinical practice. Poor absorption, short half-life, and quick elimination are some of the main reasons why the consumption of polyphenols does not result in maximal health benefits [72]. Additionally, despite fulfilling theoretical physicochemical criteria for orally bioavailable drugs, high levels of chemical modifications of natural phenolic compounds by host microbiota, along with poor water solubility of these compounds, resulting in their low bioavailability, which poses another challenge to the therapeutic application of polyphenols [73]. To overcome this poor bioavailability, new formulations are currently being developed. Recently, novel delivery systems, including nanoparticles, micelles or liposomes, are being tested. These advanced pharmacological formulations display promising amelioration of pharmacological and anticancer properties of polyphenols by optimizing pharmacokinetics and pharmacodynamics and reducing the dose needed to target tumors [72]. Finally, the approach based on combining polyphenols with conventional chemotherapeutics offers a promising avenue in oncopharmacology constituting a more efficient anticancer therapies with less side effects on human health. In this respect, further investigations of natural polyphenols are still required to decipher the most effective route of administration, the possible way of increasing their achievable concentration in vivo, and to develop novel pharmacological formulations enabling more potent drug combinations.

## Figures and Tables

**Table 1:** The basic physicochemical parameters of QCT and KMF according to the previously reported data.

Physicochemical Properties	CNS-Drugs	QCT	KMF
Molecular weight (*M*_WT_)	<450 g/mol [42]	302.23 g/mol [42,43]	286.24 g/mol [42,43]
Calculated n-octanol/water partition coefficient (*c*log*P*)	<5 [42]	~1.49 [43,44]	~1.84 [43,44]
Distribution coefficient at pH 7.4 (log*D*)	>0 < 3 [42]	0.58 [44]	0.89 [44]
Number of H-bond donor (HBD)	<3 [42]	5 [45]	4 [43,44]
Number of H-bond acceptor (HBA)	<7 [42]	7 [45]	6 [46]
Number of rotatable bonds	<8 [42]	2 [43]	4 [43]
Acid dissociation constant (p*K*a)	4–10 [42]	7.1 [47]	6.96 [47]
Polar surface area	60–90 Å^2^ [48,49]	~131.35 Å^2^ [42,44,45]	~107.21 Å^2^ [42,44]
Drug-Likeness Model Score	0–6 * [50,51]	1.64 ^#^ [42]	0.91^#^ [42]

* Evaluated by Multiparameter Optimization (MPO) method, # Calculated with Molinspiration software or free molecular property calculation services.

## Data Availability

The data presented in this study are available within the manuscript.

## Supplementary Materials

The following supporting information can be downloaded at: https://www.mdpi.com/article/10.3390/ijms251910740/s1.

## Abbreviations

BBB	Blood brain barrier
CNS	Central nervous system
ELS	Electrochemical light scattering
GBM	Glioblastoma
*ζ*	Zeta potential
logP	n-octanol/water partition coefficient
clogP	Calculated n-octanol/water partition coefficient
Ro5	Lipinski’s Rule of 5
MWT	Molecular weight
pKa	Acid dissociation constant
HBA	Hydrogen bond acceptor groups
HBD	Hydrogen bond donor groups
