# Supplementary material for: Cytotoxicity, Proapoptotic Activity and Drug-like Potential of Quercetin and Kaempferol in Glioblastoma Cells: Preclinical Insights
Source: Int J Mol Sci. 2024 Oct 5;25(19):10740. doi: 10.3390/ijms251910740 (PMC11477293; doi:10.3390/ijms251910740)
Supplement: Supplementary file 1 [file ijms-25-10740-s001.zip › ijms-3220339-supplementary.pdf]

Table S1. Effect of quercetin and kaempferol on the zeta potential values and isoelectric point values of glioblastoma T98G cell membranes.

Compound	System	Isoelectric Point	Zeta Potential Values [mV]	
			pH ~ 2	pH ~ 9
**Quercetin**	**control (24 h)**	~ 3.4	4.08 ± 0.34	-13.23 ± 0.63
	+ 25 μM	~ 3.7	3.62 ± 0.27	-10.44 ± 0.67 *
	+ 50 μM	~ 4.0	3.62 ± 0.28	-8.54 ± 0.57 *
	control (48 h)	~ 3.5	3.92 ± 0.37	-13.22 ± 0.56
	+ 25 μM	~ 3.7	3.62 ± 0.32	-10.70 ± 0.60 *
	+ 50 μM	~ 4.2	3.86 ± 0.42	-8.59 ± 0.63 *
Kaempferol	control (24 h)	~ 3.4	4.08 ± 0.34	-13.23 ± 0.63
	+ 25 μM	~ 3.5	3.90 ± 0.34	-11.65 ± 0.51 *
	+ 50 μM	~3.9	3.86 ± 0.38	-10.23 ± 0.63 *
	control (48 h)	~ 3.5	3.98 ± 0.48	-13.23 ± 0.64
	+ 25 μM	~ 3.7	4.01 ± 0.29	-11.78 ± 0.71 *
	+ 50 μM	~ 3.8	3.95 ± 0.34	-10.17 ± 0.65 *

* Statistical significance ($p \leq 0.05$).
